# Dietary Patterns and Insomnia Symptoms in Chinese Adults: The China Kadoorie Biobank

**DOI:** 10.3390/nu9030232

**Published:** 2017-03-04

**Authors:** Canqing Yu, Zumin Shi, Jun Lv, Yu Guo, Zheng Bian, Huaidong Du, Yiping Chen, Ran Tao, Ying Huang, Junshi Chen, Zhengming Chen, Liming Li

**Affiliations:** 1 Department of Epidemiology and Biostatistics, School of Public Health, Peking University Health Science Center, 38 Xueyuan Road, Beijing 100191, China; yucanqing@pku.edu.cn (C.Y.); lvjun@bjmu.edu.cn (J.L.); 2 Discipline of Medicine, University of Adelaide, SAHMRI, North Terrace, Adelaide SA 5000, Australia; 3 Chinese Academy of Medical Sciences, Fuwai Hospital Xishan Branch Court, Western Feng Cun, Mentougou, Beijing 102308, China; guoyu@kscdc.net (Y.G.); bianzheng@kscdc.net (Z.B.); 4 Nuffield Department of Population Health, University of Oxford, Richard Doll Building, Old Road Campus, Oxford OX3 7LF, UK; huaidong.du@ndph.ox.ac.uk (H.D.); yiping.chen@ndph.ox.ac.uk (Y.C.); zhengming.chen@ndph.ox.ac.uk (Z.C.); 5 Department of Non-communicable Diseases, Jiangsu Center for Disease Control and Prevention, No.172 Jiangsu Road, Gulou District, Nanjing 210009, China; trltjy@163.com; 6 Department of Non-communicable Diseases, Guangxi Center for Disease Control and Prevention, No.18 Jinzhou Road, Nanning 530028, China; huangying526@sina.com; 7 China National Center for Food Safety Risk Assessment, 37 Guangqu Road, Beijing 100738, China; chenjunshi@cfsa.net.cn

**Keywords:** dietary pattern, insomnia, sleep, difficulty initiating sleep, difficulty maintaining sleep, daytime dysfunction, Chinese

## Abstract

Limited attention has been paid to the effect of dietary patterns on sleep problems. In the present study, we analyzed the cross-sectional data of 481,242 adults aged 30–79 years from the China Kadoorie Biobank. A laptop-based questionnaire was administered to collect information on food intakes and insomnia symptoms. Logistic regression was used to estimate the odds ratios of each insomnia symptom according to quartiles of each dietary pattern, with adjustment for potential confounders. Two major dietary patterns were derived by factor analysis. The traditional northern dietary pattern was characterized by high intakes of wheat and other staple food, whereas the modern dietary pattern was characterized by high intakes of meat, poultry, fish, eggs, fresh fruit, and dairy products. Both dietary patterns were associated with a decreased prevalence of insomnia symptoms (*p* for trend < 0.001); after adjustment for potential confounders, individuals who had the highest quartile score of traditional northern dietary pattern were 12%–19% less likely to have insomnia symptoms compared to those in the lowest quartile (odds ratio: 0.81–0.88), and the corresponding values for the modern dietary pattern were 0.89–1.01. Furthermore, interactions of these two dietary patterns on insomnia symptoms were observed. Further prospective studies are needed to elucidate the relationship between diet and insomnia.

## 1. Introduction

Insomnia is one of most common sleep disorders; it is characterized by difficulties in initiating sleep, maintaining sleep, early-morning awakening, and accompanied by daytime consequences in social, occupational, behavioral, or other important areas. These symptoms were shown to be associated with mortality [1,2], cardiovascular diseases [3], and injuries [4]. 

Emerging studies have shown the relationship between dietary factors and sleep duration and sleep quality [5], but evidence on diet and insomnia symptoms is limited and unclear. A cross-sectional study among middle-aged Japanese workers showed that both a low and high intake of protein were associated with insomnia symptoms, and a low carbohydrate intake was associated with difficulty maintaining sleep [6]. A high carbohydrate intake was reported to be associated with poor sleep quality among middle-aged female Japanese workers [7]. Another study reported that the direction of association between carbohydrates and sleep quality were different due to the glycemic index of each food, such as rice, bread, and noodle [8]. Recent results from a Chinese cohort reported that dinner fat intake and soy isoflavone were associated with sleep parameters over five years [9,10]. Furthermore, intervention studies have shown that some nutrients, such as tryptophan (TRP), group B vitamins, minerals, and unrefined carbohydrates, were linked to sleep [11]. On the other hand, evidence has shown that sleep curtailment and poor sleep quality are associated with increased food consumption and poor dietary preference [12].

However, foods are consumed in combination, and it is difficult to separate the specific effects of individual foods and nutrients. Dietary patterns are the summary measures of several foods or food groups, and have gained much attention in nutritional epidemiology [13]. A healthy dietary pattern in Japanese adults—characterized by a high intake of vegetables, mushrooms, potatoes, seaweeds, soy products, and egg—was shown to be inversely associated with sleep symptoms [14]. In the present cross-sectional study, we aimed to examine the association of dietary patterns with insomnia symptoms among a large sample of Chinese adults aged 30–79 years from the China Kadoorie Biobank (CKB).

## 2. Methods 

### 2.1. Study Population 

The CKB is a general population-based prospective cohort study involving approximately 500,000 middle-aged adults in China. The detailed study design and profile are previously reported [15,16]. In brief, ten geographically diverse areas—five urban (Qingdao, Harbin, Haikou, Suzhou, and Liuzhou) and five rural (Sichuan, Gansu, Henan, Zhejiang, and Hunan)—were selected based on local patterns of disease and known risk factors, population stability, quality of death and disease registries, and local commitment and capacity. In each area, about 150 administrative units (rural villages or urban residential communities) were selected. Men and women aged 30–79 years in each community were invited to attend study assessment at clinics set up in local residential community centers. In total, 512,891 participants were recruited at a baseline survey in 2004–2008 from 1737 villages or communities in 10 study areas. Since recruitment, a random sample (~5%) of the participants in CKB has been periodically re-surveyed using largely the same procedures as the baseline. The first and second re-survey were undertaken in 2008 and 2013, respectively.

Of the 512,891 participants enrolled in the CKB baseline survey, those with a history of coronary heart diseases (*n* = 15,472), stroke (*n* = 8884), or cancer (*n* = 2577) were excluded in the present study. In addition, participants with current medication for insomnia disorder (*n* = 5455) or other psychological diseases (such as depression and anxiety, *n* = 817), were also excluded, leaving 481,424 (197,680 men and 283,744 women) available for the present analyses. Participants with a history of cardiovascular disease or cancer, or with current medication for psychological diseases, were also excluded because these conditions might affect both dietary habits and sleep symptoms. 

The study was approved by the ethical committee and research council of the Chinese Centre for Disease Control and Prevention (China, 005/2004, 8 July 2004) and the Oxford Tropical Research Ethics Committee at the University of Oxford (UK, 025-04, 3 February 2005). Prior to the survey, all participants provided written informed consent.

### 2.2. Data Collection

At the baseline survey, detailed information about general demographic and socio-economic status, dietary and other lifestyle habits (e.g., smoking, alcohol drinking, and physical activity), sleep, mental health, medical history, and current medication were collected using an interview-administered laptop-based questionnaire.

Participants’ daily physical activity was estimated as the sum of the metabolic equivalent hours per day (MET-hours/day) based on the questions on the usual type and duration of activities related to work, commuting, household chores, and leisure-time exercise during the past 12 months [17]. Depressive and anxiety symptoms were assessed using the short form of World Health Organization (WHO) 12-month Computerized International Diagnostic Interview (CIDI-SF) [18]. Body weight and body height were measured by trained technicians according to standard protocol. Body weight was measured using a TANITA TBF-300GS body composition analyzer (Tanita Corp., Tokyo, Japan) to the nearest 0.1 kg with participants wearing light clothes. Body weight was measured using a manufactured instrument to the nearest 0.1 cm, with participants standing without shoes. Body mass index (BMI) was calculated as weight in kg divided by height in m^2^. 

### 2.3. Dietary Assessment

Dietary habits during the preceding 12 months were assessed using a frequency questionnaire, covering 12 major food groups in China: rice, wheat, other staples (such as corn, millet, etc.), meat, poultry, fish, eggs, fresh fruit, fresh vegetables, preserved vegetables, soybean, and dairy products. The five frequency levels of habitual consumption (i.e., never/rarely, monthly, 1–3 days/week, 4–6 days/week, or daily), were recoded into days/week: 0, 0.5, 2, 5, and 7 respectively. At the second re-survey of the CKB study, questions on the daily intake of each food group were added to the baseline questionnaire. Hence, the weekly amount of food consumption and the daily energy intake could be estimated for each participant in the resurvey.

In addition, the frequency and quantity of beverages commonly consumed in China were also recorded, including four types of tea (green/jasmine tea, oolong tea, black tea, or other tea) and five types of alcohol (beer, rice wine, wine, spirit with ≥40% alcohol, or spirit with <40% alcohol). Thus, the average consumption (in g/week) was calculated [19].

A repeated questionnaire survey among 926 participants was performed within one year after the baseline, and showed good reproducibility of the food and beverage questionnaire (Supplemental Table S1).

### 2.4. Definition of Outcome

The CKB baseline survey constructed three insomnia symptom questions based on the guidelines of the *Diagnostic and Statistical Manual of mental disorders 5th edition* (DSM-5) by the American Psychiatry Association. Participants reported their sleep symptoms on three questions during the last month for at least 3 days each week: (1) “taking >30 min to fall asleep after going to bed or waking up in the middle of night?” (difficulty initiating or maintaining sleep, DIMS); (2) “Waking up early and not being able to go back to sleep?” (early-morning awakenings, EMA); and (3) “Having difficulty staying alert while at work, eating or meeting people during daytime” (daytime dysfunction, DDF). The response options for each question were dichotomous (yes or no). A good reliability was shown among the 926 individuals who had a repeated questionnaire survey within one year after the baseline, all agreements were >0.80, and Prevalence and Bias Adjusted Kappa (PABAK) [20,21] were >0.60.

### 2.5. Statistical Analysis

Dietary patterns were derived from the twelve food groups and nine beverage groups using factor analysis with a principal component method. The factors were rotated by orthogonal transformation (varimax rotation) to maintain the independent factors and greater interpretability. The number of factors (dietary patterns) was determined based on eigenvalue (>1), scree plot, factor interpretability, and the variance explained (5%) by each factor. The dietary patterns were named according to the food groups showing high loadings (absolute value) on each factor. The factor scores for each dietary pattern and each participant were calculated by summing the consumption of each food group that was weighted by its factor loading. 

The participants were divided into quartiles according to the dietary pattern score (Q1 to Q4 in ascending order). The characteristics were expressed as the mean and percentages for continuous and categorical variables, respectively. Trend association was assessed by assigning median dietary pattern score to each quartile, and was tested using a linear regression analysis or the Cochrane–Armitage trend test for continuous and categorical variables, respectively. Logistic regression models were performed to estimate the odds ratios and 95% confidence interval (95% CI) for each outcome according to the quartile of each dietary pattern, and the lowest quartile was used as the reference category. Model 1 was adjusted for age (continuous, year), gender, and study area. Model 2 was additionally adjusted for BMI (continuous, kg/m^2^), married (yes or no), manual work (yes or no), education attainment (no formal school, primary school, middle school, high school, or college/university), household income (<10,000, 10,000–19,999, ≥20,000 Chinese yuan (CNY)/year), physical activity (<13.0, 13.0 to 26.0, >26.1 MET-h/day), smoking status (never/occasional, former, current and 1 to 14 cigarettes/day, current and 15 to 24 cigarettes/day, or current and ≥25 cigarettes/day), alcohol consumption (not weekly drinking, ex-regular drinkers, not daily, daily and <15 g/day, daily and 15–29 g/day, daily and 30–59 g/day, or daily and ≥60 g/day), hypertension (yes or no), diabetes (yes or no), depressive symptom (yes or no), and anxiety symptom (yes or no). A test for linear trend across quartiles in the logistic models was performed by assigning a median value to each quartile of each dietary pattern, producing a single continuous variable used to model the *P* trend, and odds ratio (OR) and 95% confidence interval (95% CI) for 1 standard deviation (1-SD) increase was estimated for each dietary pattern. We tested the multiplicative interaction of derived dietary patterns by likelihood ratio tests to compare the difference between models with and without the interaction term, and then estimated the joint effect of derived dietary patterns on each insomnia problem.

In the sensitive analysis, we used the data from the second re-survey, which collected information on frequency and daily intake of all 12 food groups among 24,996 (~5%) randomly selected participants to estimate the daily consumption amount by study area, gender, and age. Dietary patterns were then derived using the same method and were compared with the results of our primary analysis. Furthermore, daily energy intake for each participant at baseline was calculated and was additionally adjusted based on the second multivariable model in its log-transformed form. In addition, those who had a medical history of hypertension (*n* = 47,306), diabetes (*n* = 12,860), or any depressive or anxiety symptoms (*n* = 14,242) were excluded because these complications might affect both dietary habits and sleep symptoms. All statistical analyses were performed with SAS version 9.3 (SAS Institute Inc., Cary, NC, USA). Significance was defined as *p* < 0.05.

## 3. Results 

Two dietary patterns were identified (Table 1). The first pattern was characterized by low intakes of rice and high intakes of wheat and other staple foods like corn and millet, and was named as the traditional northern dietary pattern. Participants with the higher score in this dietary pattern followed a typical traditional northern dietary pattern. On the other hand, participants with low dietary pattern score who intake rice as staple food followed a typical Southern dietary pattern (Supplemental Table S2). The second pattern was characterized by high intakes of meat, poultry, fish, eggs, fresh fruit, and dairy products, and was named as the modern dietary pattern. These two dietary patterns explained 24.3% of the variability. The same dietary patterns were derived using the same method from the second re-survey data which collected the information on both frequency and amount. About 75.7% and 71.3% of participants were categorized into the same quartile, and their weighted kappa were 0.80 and 0.77 for the two dietary patterns, respectively (Supplemental Table S3).

Compared to participants with a lower score of traditional northern dietary pattern, those with higher scores were younger, from a northern area, and with higher education, but lower annual household income. For modern dietary pattern, the participants with higher scores were younger, more likely to be male, from southern and urban area, married, with higher education level and annual household income, and more likely to report be a drinker of alcohol, having higher reported hypertension or diabetes, but less likely to report depression or anxiety symptoms in last 12-month (Table 2).

The prevalence of DIMS, EMA, and DDF was 10.5%, 9.7%, and 1.8%, respectively. The ORs and 95% CIs of sleep symptoms according to the quartile of each dietary pattern score are presented in Table 3. Compared with the lowest quartile in the traditional northern dietary pattern, the highest quartile had a significantly lower likelihood of sleep problems (OR, 95% CI for DIMS: 0.81, 0.76–0.86; EMA: 0.88, 0.83–0.93; DDF: 0.85, 0.75–0.96; All *p* for trend <0.05). In addition, a 1-SD increase in dietary scores was associated with a 3–7% lower risk of sleep problems (OR, 95% CI for DIMS: 0.93, 0.91–0.95; EMA: 0.97, 0.95–1.00; DDF: 0.94, 0.89–0.99). Similar results were observed in the modern dietary pattern for sleep problems of DIMS and EMA, but not for DDF (DIMS: 0.89, 0.86–0.93; EMA: 0.93, 0.90–0.97; DDF: 0.93, 0.90–1.10; all *p* for trend <0.05 except for 0.72 for DDF). A 1-SD increase in the modern dietary pattern was significantly associated with a 5% and 3% lower risk of DIMS and EMA, respectively. The associations remained after additional adjustment for estimated daily energy intake in the multivariable logistic model (Supplemental Table S4). 

**Figure 1:**
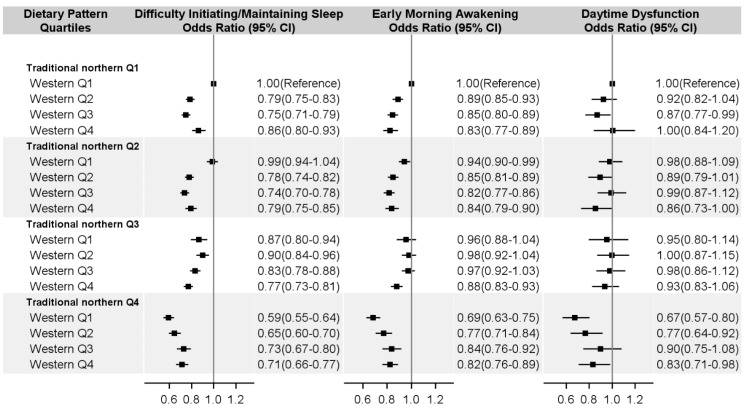
Odds ratio and 95% confidence interval of sleep problems according to the joint classification of dietary patterns. Note: Multivariable-adjusted odds ratio estimated from logistic models adjusted for study site, age (year), gender, BMI (kg/m^2^), married (yes or no), manual work (yes or no), education attainment (no formal school, primary school, middle school, high school, or college/university), household income (<10,000, 10,000–19,999, ≥20,000 CNY/year), physical activity (<13.0, 13.0 to 26.0, >26.1 MET-h/day), smoking status (never/occasional, former, current and 1 to 14 cigarettes/day, current and 15 to 24 cigarettes/day, or current and ≥25 cigarettes/day), alcohol consumption (not weekly drinking, ex-regular drinkers, not daily, daily and <15 g/day, daily and 15–29 g/day, daily and 30–59 g/day, or daily and ≥60 g/day), hypertension (yes or no), diabetes (yes or no), depressive symptom (yes or no), and anxiety symptom (yes or no). All *p* values for interaction were < 0.001. Q: quartile; CI: Confidence Interval; BMI: body mass index; CNY: Chinese yuan; MET-h/day: metabolic equivalent hours per day.

The joint analysis using the traditional northern and modern dietary pattern scores by quartiles showed that compared with participants who had the lowest score in both dietary patterns, those in highest scores in traditional northern dietary pattern and lowest scores in the modern dietary pattern had the lowest risk of sleep symptoms (DIMS: 0.59, 0.55–0.64; EMA: 0.69, 0.63–0.75; DDF: 0.67 0.57–0.80; all *p* for interaction < 0.05) (Figure 1).

In sensitivity analyses, after the participants with mental disorders (depression and anxiety) and vascular insults (diabetes and hypertension) were excluded, both the association and linear trend between dietary patterns with sleep problems remained (Supplemental Table S5).

## 4. Discussion 

Two major dietary patterns were identified in the present analysis of a cross-sectional study in a large sample of Chinese adults. The traditional northern dietary pattern—characterized by high intakes of wheat and other staple food—was associated with a decreased prevalence of DIMS, EMA, and DDF. The modern dietary pattern—characterized by high intakes of meat, poultry, fish, eggs, fresh fruit, and dairy products—was associated with a decreased prevalence of DIMS and EMA. These associations were dose-dependent, and persisted after further adjustment for daily energy intake and further exclusion of participants with vascular insults or mental disorders.

The dietary patterns constructed in the present study were based on the foods and beverages consumed in China, and they fully captured the characteristics of the Chinese diet and its nutrition transition due to socio-economic and cultural changes in recent decades, and were consistent with previously reported results from a large-scale nationwide survey of Chinese adults using a validated semi-quantitative food frequency questionnaire [22] and a three-day food record [23].

The traditional northern dietary pattern score was strongly correlated with a low intake of rice but high intakes of wheat and other grains as staple food, and was associated with a decreased risk of insomnia symptoms. The present results are in line with a study of sleep quality among rural elderly in China, in which rice as major staple food was a significant predictor of poor sleep quality [24]. Possible mechanisms of the effect may be related to the higher group B vitamins and minerals in wheat products and other staple food than in rice. Niacin (vitamin B_3_) and pyridoxine (vitamin B_6_) may promote the availability of TRP for the synthesis of neurotransmitter serotonin and the neurosecretory hormone melatonin, and hence improve the sleep onset latency and sleep quality [11]. Magnesium could enhance the secretion of melatonin by stimulating serotonin *N*-acetyltransferase activity [25], as well as act as a γ-aminobutyric acid agonist to improve sleep [26]. However, inconsistent results were reported in a cross-sectional study among Japanese adults [8]. Therefore, further studies on the associations and their underlying mechanisms between long-term consumption of starchy staple food and sleep quality are needed.

In the present study, the modern dietary pattern score was strongly correlated with high intakes of high-protein food such as meat, poultry, fish, and eggs, and was associated with a low prevalence of all three insomnia symptoms. Existing evidence has shown that dietary protein intake was inversely associated with insomnia [27], and higher protein intake may improve sleep–wake regularity and sleep quality [28,29]. The mechanism of the effect of protein on sleep may be related to TRP, which is a precursor to serotonin and melatonin, which regulate the sleep cycle [30]. Vitamin B_12_—which is abundant in the modern dietary pattern—could contribute to melatonin secretion by enhancing the receptors in the brain [31].

Moreover, some foods in the modern dietary pattern, such as dairy products, fatty fish, and fresh fruit also have been reported sleep-promoting effect [32]. Fatty fish—known as a good source of omega-3 fatty acids and vitamin D—was positively associated with sleep efficiency and sleep quality in previous studies [33,34]. Fresh fruit with high antioxidant capacity and folate content may also minimize the oxidative damage of sleep and psychiatric disorders [35]. 

Of note, we observed that participants who had the highest intake of wheat and other staple but lowest rice, the modern dietary pattern score was associated with increased risks of sleep problems. One possible explanation may be linked to the ratio of plasma TRP to large-chain neutral amino acids (LCAANs), which enter the brain in a competitive manner with TRP. It is known that the ratio is affected by both dietary carbohydrates and dietary proteins [36]. A high carbohydrate diet that is low in protein has been shown to elevate brain TRP concentrations relative to higher-protein diets in animal models [37]. Furthermore, the consumption of carbohydrates with high glycemic index—such as white rice in the traditional northern dietary pattern—could trigger the secretion of insulin, and enhances the TRP-to-LCAAN ratio by facilitating uptake of LCAANs by muscle, therefore promoting TRP entry into the brain and upregulating serotonin production [38]. This proposed mechanism is yet to be determined and must be explored further.

The present study is the first and the largest study of the influence of dietary pattern on sleep problems in a Chinese population. Limitations of the present study also warrant mention. First of all, we only assessed 12 crude food groups that are commonly consumed in the Chinese diet; other food groups that may be related to sleep problems were not addressed. Second, the tools used to collect the information on food intake were non-validated FFQ. We only collected frequencies of each food consumption, without food quantity. However, according to results from the second CKB re-survey with food frequency questionnaire, the dietary pattern score using food frequency only was highly consistent with those using both food frequency and amount. Third, the method of constructing dietary pattern requires subjective choice in determining the number of factors as well as naming the dietary patterns. However, the dietary patterns constructed in the present study captured the most typical diet habits in the Chinese population, and counterparts have been observed in previous Chinese studies. Fourth, the measurement of sleep problems were cross-sectional and self-reported, and the questions were not in complete compliance with clinical diagnostic standard (i.e., DSM-5), though insomnia symptoms in the present study were strictly defined with the minimum frequency of three days per week during the past month. Fifth, an association derived from a cross-sectional study cannot address causality or the direction of the relation. Hence, a possibility of the reciprocal influence of sleep disturbances on dietary patterns cannot be excluded. Sixth, although we excluded participants with chronic conditions and adjusted for a series of covariates, the possibility of residual confounding and unknown confounders cannot be excluded.

## 5. Conclusions

In conclusion, the traditional northern and modern dietary patterns—which are the major dietary patterns in the Chinese population—were associated with lower sleep problems in adults. The associations observed in the cross-sectional observational study need further investigation in prospective studies.

## Figures and Tables

**Table 1:** Factor loading matrix of major dietary patterns by principal component analysis with varimax rotation (*n* = 481,424).

Food or Beverage Group	Traditional Northern Dietary Pattern	Modern Dietary Pattern
Rice	**−0.84**	0.20
Wheat	**0.84**	0.12
Other staple	**0.70**	−0.16
Meat	−0.36	**0.60**
Poultry	−0.35	**0.55**
Fish	−0.35	**0.52**
Eggs	0.32	**0.51**
Fresh vegetables	−0.06	0.20
Soybean	−0.12	**0.47**
Preserved vegetables	−0.15	0.13
Fresh fruit	0.03	**0.69**
Dairy products	0.23	**0.63**
Beer	0.06	0.20
Rice wine	−0.14	<0.01
Wine	<0.01	0.06
Heavy spirit (≥40%)	−0.08	<0.01
Light spirit (<40%)	−0.11	−0.02
Green tea	<0.01	0.23
Oolong tea	−0.08	0.07
Black tea	−0.19	−0.04
Other tea	−0.01	0.01
Variance explained (%)	14.58	9.74

Figures in bold indicate absolute factor loading are more than 0.40.

**Table 2:** Characteristics of participants by quartile categories of each dietary pattern score (*n* = 481,424).

Characteristics	Traditional Northern Dietary Pattern			Modern Dietary Pattern		
	Q1	Q4	*p* for Trend	Q1	Q4	*p* for Trend
Age, years	51.5	50.2	<0.0001	51.7	50.5	<0.0001
Female, %	55.2	58.8	<0.0001	64.5	57.4	<0.0001
Urban area, %	47.9	20.4	<0.0001	8.1	83.4	<0.0001
Southern area, %	98.8	0.5	<0.0001	48.2	51.3	<0.0001
Married, %	91.9	91.6	0.3487	88.7	92.0	<0.0001
High school and above, %	36.4	49.7	<0.0001	29.6	77.4	<0.0001
Household income ≥20,000 CNY/year, %	56.1	19.4	<0.0001	16.9	64.8	<0.0001
Manual worker, %	58.9	73.6	<0.0001	83.2	31.1	<0.0001
Current drinker, %	21.8	10.4	<0.0001	9.9	19.9	<0.0001
Current smoker, %	31.6	26.0	<0.0001	25.1	24.6	0.2328
Physical activity, MET-h/day	24.4	21.6	<0.0001	22.6	19.0	<0.0001
Depressive symptom, %	3.0	3.0	0.0049	4.1	1.6	<0.0001
Anxiety symptom, %	0.5	0.7	0.0001	0.8	0.3	<0.0001
Hypertension, %	10.5	7.9	<0.0001	8.3	11.3	<0.0001
Diabetes, %	2.1	2.6	<0.0001	1.8	4.0	<0.0001
BMI, kg/m^2^	23.4	23.9	<0.0001	23.2	24.3	<0.0001
Sleep duration, h	7.4	7.7	<0.0001	7.4	7.3	<0.0001
Energy intake, kcal/day	1287	1372	<0.0001	1151	1539	<0.0001

Abbreviations: CNY: unit of Chinese money Yuan; MET: metabolic equivalent; BMI: body mass index; Q: quartile. *p* for trend is based on Cochran–Armitage trend test for categorical variables and linear regression analysis for continuous variables, assigning median values to quartile categories of each dietary pattern.

**Table 3:** Odds ratios and 95% confidence interval of sleep problems by quartile of dietary pattern score.

	Quartile of Dietary Pattern Scores					OR Equivalent to 1-SD Increase
	Q1 (Low)	Q2	Q3	Q4 (High)	*p* for Trend	
**Traditional northern dietary pattern**						
Difficulty initiating/maintaining sleep						
Number of cases (%)	12,217 (10.2)	13,626 (11.3)	11,703 (9.7)	13,042 (10.8)		
Model 1	1.00 (Reference)	0.98 (0.95–1.00)	0.95 (0.92–0.98)	0.77 (0.73–0.81)	<0.0001	0.91 (0.89–0.93)
Model 2	1.00 (Reference)	0.99 (0.96–1.02)	0.99 (0.96–1.03)	0.81 (0.76–0.86)	<0.0001	0.93 (0.91–0.95)
Early morning awakening						
Number of cases (%)	12,691 (10.5)	13,360 (11.1)	11,429 (9.5)	9433 (7.8)		
Model 1	1.00 (Reference)	0.94 (0.91–0.96)	0.96 (0.93–0.99)	0.79 (0.75–0.84)	<0.0001	0.93 (0.91–0.95)
Model 2	1.00 (Reference)	0.96 (0.93–0.99)	1.05 (1.01–1.08)	0.88 (0.83–0.93)	0.0175	0.97 (0.95–1.00)
Daytime dysfunction						
Number of cases (%)	1897 (1.6)	2280 (1.9)	2092 (1.7)	2613 (2.2)		
Model 1	1.00 (Reference)	1.00 (0.94–1.07)	1.01 (0.93–1.09)	0.83 (0.73–0.93)	0.004	0.93 (0.89–0.98)
Model 2	1.00 (Reference)	1.00 (0.93–1.07)	1.03 (0.95–1.11)	0.85 (0.75–0.96)	0.0135	0.94 (0.89–0.99)
**Modern dietary pattern**						
Difficulty initiating/maintaining sleep						
Number of cases (%)	16,188 (13.5)	12,690 (10.5)	11,244 (9.3)	10,466 (8.7)		
Model 1	1.00 (Reference)	0.88 (0.86–0.91)	0.82 (0.79–0.84)	0.78 (0.75–0.81)	<0.0001	0.90 (0.89–0.91)
Model 2	1.00 (Reference)	0.92 (0.89–0.94)	0.89 (0.86–0.91)	0.89 (0.86–0.93)	<0.0001	0.95 (0.94–0.97)
Early morning awakening						
Number of cases (%)	14,016 (11.7)	12,483 (10.4)	11,105 (9.2)	9309 (7.7)		
Model 1	1.00 (Reference)	0.91 (0.89–0.94)	0.84 (0.82–0.87)	0.75 (0.73–0.78)	<0.0001	0.89 (0.87–0.90)
Model 2	1.00 (Reference)	0.98 (0.95–1.01)	0.96 (0.93–0.99)	0.93 (0.90–0.97)	0.0002	0.97 (0.96–0.99)
Daytime dysfunction						
Number of cases (%)	3174 (2.6)	2196 (1.8)	1831 (1.5)	1681 (1.4)		
Model 1	1.00 (Reference)	0.97 (0.91–1.02)	0.94 (0.88–1.00)	0.83 (0.77–0.90)	<0.0001	0.93 (0.90–0.96)
Model 2	1.00 (Reference)	1.02 (0.96–1.08)	1.05 (0.98–1.13)	1.01 (0.93–1.10)	0.7166	1.01 (0.97–1.04)

Model 1: adjusted for site, age (year), and gender. Model 2: Model 1 + BMI (kg/m^2^), married (yes or no), manual work (yes or no), education attainment (no formal school, primary school, middle school, high school, or college/university), household income (<10,000, 10,000–19,999, ≥20,000 CNY/year), physical activity (<13.0, 13.0 to 26.0, >26.1 MET-h/day), smoking status (never/occasional, former, current and 1 to 14 cigarettes/day, current and 15 to 24 cigarettes/day, or current and ≥25 cigarettes/day), alcohol consumption (not weekly drinking, ex-regular drinkers, not daily, daily and <15 g/day, daily and 15–29 g/day, daily and 30–59 g/day, or daily and ≥60 g/day), hypertension (yes or no), diabetes (yes or no), depressive symptom (yes or no), and anxiety symptom (yes or no). OR: odds ratio; Q: quartile; SD: standard deviation; BMI: body mass index; CNY: Chinese yuan; MET-h/day: metabolic equivalent hours per day.

## Supplementary Materials

The following are available online at http://www.mdpi.com/2072-6643/9/3/232/s1.
