# Supplementary material for: Dietary Patterns and Insomnia Symptoms in Chinese Adults: The China Kadoorie Biobank
Source: Nutrients. 2017 Mar 4;9(3):232. doi: 10.3390/nu9030232 (PMC5372895; doi:10.3390/nu9030232)
Supplement: Supplementary file 1 [file nutrients-09-00232-s001.pdf]

Supplemental Table 1. Spearman correlation coefficients comparing food intake at baseline with a repeat questionnaire within one year (n = 926).

Food or beverage group	Crude	Age-, sex-adjusted
Rice	0.75	0.74
Wheat	0.76	0.76
Other staple foods	0.45	0.44
Meat	0.48	0.48
Poultry	0.48	0.48
Fish	0.60	0.60
Eggs	0.58	0.58
Fresh vegetables	0.17	0.16
Soybean	0.35	0.35
Preserved vegetables	0.54	0.54
Fresh fruit	0.55	0.54
Dairy products	0.66	0.66
Beer	0.31	0.27
Rice wine	0.43	0.42
Wine	0.40	0.40
Heavy spirit (≥40%)	0.64	0.60
Light spirit (<40%)	0.40	0.38
Green tea	0.71	0.69
Oolong tea	0.72	0.71
Black tea	0.63	0.63
Other tea [1]	-	-

[1] Data is singular and kappa = 1.

Supplemental Table 2. Characteristics of dietary patterns by quartile categories.

Food or beverage group	Traditional northern dietary pattern				Modern dietary pattern				Overall Mean (Standard deviation)
	Q1	Q2	Q3	Q4	Q1	Q2	Q3	Q4	
**Food group, day/week**									
Rice	7.0	7.0	5.8	1.6	4.0	5.5	6.1	5.7	5.3 (2.6)
Wheat	0.9	1.6	5.2	7.0	4.0	3.0	3.2	4.5	3.7 (2.9)
Other staple	0.3	0.5	0.9	4.0	2.4	1.3	0.8	1.1	1.4 (2.3)
Meat	4.6	3.8	4.5	2.0	1.5	3.1	4.6	5.7	3.7 (2.5)
Poultry	2.0	1.4	1.6	0.4	0.2	1.0	1.8	2.4	1.4 (1.6)
Fish	2.0	1.4	1.6	0.4	0.2	1.0	1.8	2.4	1.4 (1.6)
Eggs	1.3	2.2	3.3	3.2	1.1	2.3	2.6	4.0	2.5 (2.2)
Fresh vegetables	6.9	6.9	6.9	6.7	6.6	6.9	6.9	7.0	6.8 (0.8)
Soybean	1.8	1.7	2.1	1.2	0.7	1.5	2.0	2.6	1.7 (1.6)
Preserved vegetables	2.7	2.0	2.4	1.6	1.7	2.1	2.3	2.5	2.2 (2.4)
Fresh fruit	1.9	2.5	3.7	2.1	0.8	1.5	2.7	5.2	2.6 (2.5)
Dairy products	0.1	0.5	1.9	1.3	<0.1	0.2	0.6	3.0	1.0 (2.1)
**Beverage group, g/week**									
Beer	1.0	2.0	8.3	5.3	0.1	0.7	2.5	13.2	4.1 (34.0)
Rice wine	12.2	1.1	0.4	<0.1	1.6	4.4	5.1	2.6	3.4 (35.1)

Wine	0.1	0.1	0.2	0.1	<0.1	<0.1	0.1	0.4	0.1 (3.8)
Heavy spirit (≥40%)	37.5	23.6	23.9	11.8	21.8	26.6	26.0	22.3	24.2 (112.6)
Light spirit (<40%)	37.5	23.6	23.9	11.8	21.8	26.6	26.0	22.3	24.2 (112.6)
Green tea	4.9	6.7	9.0	5.6	2.6	5.2	7.3	11.1	6.6 (16.0)
Oolong tea	0.9	0.4	0.2	<0.1	<0.1	0.2	0.5	0.8	0.4 (4.5)
Black tea	3.6	0.4	0.1	<0.1	1.2	1.5	1.0	0.5	1.0 (7.6)
Other tea	<0.1	<0.1	<0.1	<0.1	<0.1	<0.1	<0.1	<0.1	0.0 (0.7)

Supplemental Table 3. Comparing dietary patterns constructed from food consumption frequency and estimated amount. [1]

Traditional northern dietary pattern [2]

		Estimated food consumption amount				Total	kappa	Weighted kappa
		Q4	Q3	Q2	Q1			
Food consumption frequency	Q1	91,445 (19.0)	28,381 (5.9)	528 (0.1)	0 (0.0)	120,354	0.675	0.800
	Q2	25,949 (5.4)	72,914 (15.2)	21,495 (4.5)	0 (0.0)	120,358		
	Q3	2962 (0.6)	19,054 (4.0)	88,932 (18.5)	9409 (2.0)	120,357		
	Q4	0 (0.0)	2 (<0.1)	9406 (2.0)	110,947 (23.1)	120,355		
	Total	120,356	120,351	120,361	120,356	481,424		

Modern dietary pattern

		Estimated food consumption amount				Total	kappa	Weighted kappa
		Q1	Q2	Q3	Q4			
Food consumption frequency	Q1	94,180 (19.6)	25,979 (5.4)	197 (<0.1)	0 (0.0)	120,356	0.618	0.769
	Q2	25,464 (5.3)	68,247 (14.2)	26,579 (5.5)	65 (<0.1)	120,355		
	Q3	710 (0.2)	25,929 (5.4)	77,232 (16.0)	16,486 (3.4)	120,357		
	Q4	2 (<0.1)	201 (<0.1)	16,348 (3.4)	103,805 (21.6)	120,356		
	Total	120,356	120,356	120,356	120,356	481,424		

[1] Data in the table are shown as frequencies and overall percentage. [2] The quartile of a traditional northern dietary pattern of estimated amount were reversed to match the same direction of the food groups.

Supplemental Table 4. Odds ratios and 95% confidence intervals of sleep problems by quartile of dietary pattern score with additional adjustment of daily energy intake.

	Quartile of dietary pattern scores				
	**Q1**	**Q2**	**Q3**	**Q4**	***p* for trend**
**Traditional northern dietary pattern**					
Difficulty initiating/maintaining sleep	1.00 (Reference)	0.99 (0.96–1.02)	1.02 (0.98–1.05)	0.83 (0.79–0.88)	<0.0001
Early morning awakening	1.00 (Reference)	0.96 (0.93–0.99)	1.03 (0.99–1.07)	0.86 (0.81–0.91)	0.0003
Daytime dysfunction	1.00 (Reference)	1.00 (0.93–1.06)	0.98 (0.90–1.07)	0.80 (0.70–0.91)	0.0007
**Modern dietary pattern**					
Difficulty initiating/maintaining sleep	1.00 (Reference)	0.92 (0.89–0.94)	0.89 (0.85–0.92)	0.89 (0.85–0.94)	<0.0001

Early morning awakening	1.00 (Reference)	0.94 (0.92–0.97)	0.89 (0.86–0.93)	0.82 (0.78–0.87)	<0.0001
Daytime dysfunction	1.00 (Reference)	0.99 (0.93–1.05)	0.99 (0.92–1.07)	0.91 (0.81–1.01)	0.0848

Odds ratios and 95% confidence intervals are adjusted for study site, age (year), gender, BMI (kg/m²), married (yes or no), manual work (yes or no), education attainment (no formal school, primary school, middle school, high school, or college/university), household income (<10,000, 10,000–19,999, ⩾ 20,000 CNY/year), physical activity (<13.0, 13.0 to 26.0, >26.1 MET-h/day), smoking status (never/occasional, former, current and 1 to 14 cigarettes/day, current and 15 to 24 cigarettes/day, or current and ⩾25 cigarettes/day), alcohol consumption (not weekly drinking, ex-regular drinkers, not daily, daily and <15 g/day, daily and 15–29 g/day, daily and 30–59 g/day, or daily and ⩾60 g/day), hypertension (yes or no), diabetes (yes or no), depressive symptom (yes or no), anxiety symptom (yes or no), and log-transformed total energy intake (kcal/day).

Supplemental Table 5. Odds ratios and 95% confidence intervals of sleep problems by quartile of dietary pattern score among participants without mental symptoms or somatic diseases (*n* = 412,830).

	Quartile of dietary pattern scores				
	**Q1**	**Q2**	**Q3**	**Q4**	***p* for trend**
**Traditional northern dietary pattern**					
Difficulty initiating/maintaining sleep	1.00 (Reference)	0.98 (0.95–1.01)	0.99 (0.95–1.03)	0.79 (0.74–0.84)	<0.0001
Early morning awakening	1.00 (Reference)	0.95 (0.92–0.98)	1.04 (1.00–1.08)	0.85 (0.80–0.91)	0.0039
Daytime dysfunction	1.00 (Reference)	0.97 (0.90–1.05)	0.98 (0.89–1.08)	0.84 (0.73–0.97)	0.0306
**Modern dietary pattern**					
Difficulty initiating/maintaining sleep	1.00 (Reference)	0.92 (0.90–0.95)	0.90 (0.87–0.93)	0.91 (0.87–0.94)	<0.0001
Early morning awakening	1.00 (Reference)	0.99 (0.96–1.02)	0.97 (0.93–1.00)	0.95 (0.91–0.99)	0.0081
Daytime dysfunction	1.00 (Reference)	1.03 (0.97–1.11)	1.07 (0.98–1.15)	1.03 (0.94–1.14)	0.4593

Odds ratios and 95% confidence intervals are adjusted for site, age (year), gender, BMI (kg/m²), married (yes or no), manual work (yes or no), education attainment (no formal school, primary school, middle school, high school, or college/university), household income (<10,000, 10,000–19,999, ⩾ 20,000 CNY/year), physical activity (<13.0, 13.0 to 26.0, >26.1 MET-h/day), smoking status (never/occasional, former, current and 1 to 14 cigarettes/day, current and 15 to 24 cigarettes/day, or current and ⩾25 cigarettes/day), and alcohol consumption (not weekly drinking, ex-regular drinkers, not daily, daily and <15 g/day, daily and 15–29 g/day, daily and 30–59 g/day, or daily and ⩾60 g/day).
